# Invisible Agents of Rural Development. Russian Immigrant Women in the Finnish Border Region

**DOI:** 10.3389/fsoc.2021.601726

**Published:** 2021-06-25

**Authors:** Maarit Sireni, Pirjo Pöllänen, Olga Davydova-Minguet

**Affiliations:** Karelian Institute, University of Eastern Finland, Joensuu, Finland

**Keywords:** Finnish-Russian border, immigration, women, transnationalism, vitality, resilience

## Abstract

The rural region of North Karelia is located close to the Finnish-Russian border, and faces challenges due to population decline and labor shortage. However, it has a unique strength which is its proximity to Russia. This paper analyses the perceived role of immigration in enhancing the vitality of North Karelia. It investigates how the impact of immigration is presented in the regional media, and how Russian-speaking immigrant women’s roles as “agents of development” can be interpreted on the basis of their interviews. The analysis is based on text material obtained from the regional newspaper, and ethnographic interviews conducted among Russian-speaking immigrant women. Findings based on the newspaper material indicate that immigrants are valued primarily for their contribution to regional economic development. However, in some of the texts, immigrants are presented as an integral part of the region’s population, who diversify the skills of communities and thus create potential for promoting local resilience. The analysis of the interview data indicates that the proximity of the border, transnational connections, and ethno-cultural capital which is based on immigrants’ national background are important factors that impact on the attractiveness of North Karelia for Russian immigrant women. Everyday transnational multiculturalism encompasses women’s precarious employment which impacts on the well-being of broader communities on both sides of the border. Although Russian immigrant women are a vital part of these communities, they do not themselves participate in the newspaper discussions about the vitality of rural communities. This indicates that Russian women are “invisible” agents of rural development, who are not fully recognized as contributors of resilience in North Karelia.

## Introduction

### Aims

Recent patterns of migration in Europe indicate that in addition to well-established urban destinations, an increasing number of international migrants are attracted by rural areas (de Lima et al., 2005; McAreavey 2012). This is largely due to a demand for inexpensive and flexible labor in agriculture, food processing, construction, and tourism industries (Rye and Andrzejewska 2010; Dufty-Jones 2014; Bock et al., 2016; Rye and Scott 2018). Other motives of international migrants who end up in rural areas relate to lifestyle reasons of those who seek a rural idyll (Krivokapic-Skoko and Collins 2014) or pursue other personal goals (Carson and Carson 2018). Among the multiple causes of migration, marriage-based migration has been seen to lead to increased border crossings and a globalization of the countryside (Flemmen and Lotherington 2008; Pöllänen 2013).

Within rural studies, immigration is identified as an asset to rural locations which suffer from labor shortage and population decline (Kasimis et al., 2010). When immigrants are integrated in rural communities, they can also contribute to local resilience–i.e. the capacity of rural communities to cope with, adjust to, and recover from economic and social transformations (McManus et al., 2012; Kotilainen et al., 2015; Søholt et al., 2018). Though it is hoped that immigrants may be a means by which to “rescue” rural regions (Aure et al., 2018), research suggests that foreign arrivals are not always included in rural communities (Søholt et al., 2018). Immigrants in destinations with a limited experience of immigration may face resistance (McConnell and Miraftab 2009) and find themselves trapped in low-paid precarious positions (Rye and Scott 2018), as rural areas often lack the services, networks and competencies to effectively support their integration (McAreavey and Krivokapic-Skoko 2019).

Within migration studies, the concept of integration has been defined and approached from various perspectives (Saukkonen 2020; Martiniello and Rath 2014). Though immigrants would not meet the criteria of integration as defined above (e.g., labor market integration, language skills), their own everyday experiences of integration may be different (Saukkonen, 2020, see also; Könönen, 2015). The concept of transnationalism challenges the traditional integration discussion because of its methodological nationalism. Methodological nationalism has been criticized for the way of seeing a social phenomenon only through the lens of the nation state. The transnational perspective concentrates on the everyday reality of immigrants, which is constructed and bodily lived in more than one location through social, economic, political and cultural ties with their places of origin (Levitt and Glick Schiller 2004; Assmuth et al., 2018). For example, rural border areas provide some individuals, such as women who are responsible for giving care on the other side of the border, with a livable context for their transnational everyday lives (Pöllänen and Davydova-Minguet 2017).

The present article combines the perspectives of rural studies and migration studies, and analyzes the role of immigration in rural development in a new immigration destination in Finland. Firstly, it investigates the interpretations of the role of immigrants in enhancing the vitality of the region North Karelia in the Finnish-Russian border area in eastern Finland by analyzing how the impact of immigration is presented in the regional newspaper. Secondly, the study analyzes how the border region’s vitality can be perceived through the interviews of Russian immigrant women who live their transnational everyday lives in the rural border area*.* The analysis presented in this study is based on two qualitative data sets: text material obtained from the regional newspaper, and ethnographic interviews conducted among Russian-speaking immigrant women. Combining these two approaches, the study explores how the public discussion frames the everyday lives of female immigrants, and how they negotiate and contest the roles that are offered to them in their immigration destination.

In the following section, the region of North Karelia, the Finnish vitality policy and resilience, and the concepts of integration and transnationalism are introduced. Data sets and analysis methods are presented in Materials and Methods. Findings are presented in Results, starting with the findings of the media discussion, and proceeding to the findings of the ethnographic research. The discussion section concludes the paper.

### Context: North Karelia

The region of North Karelia, located in eastern Finland, represents a predominantly rural and remote area within the EU (ESPON 2011, p. 17). Amongst other European NSPA (northern sparsely populated areas) regions, North Karelia has unique geographical characteristics–a sparse, declining, and aging population, a harsh climate, abundant natural resources, a relative lack of agriculture, a strong potential for renewable energy, long distances from markets, and high costs of land transport. According to the (OECD 2017, p. 20), its strengths in the Finnish economy include its forestry resources (wood and minerals exports), environmental assets (wilderness areas), and its proximity to Russia. North Karelia, which features a 304 km long border with Russia, is part of the European gateway to the east of the Russian Federation. It has the relatively prominent checkpoint of Niirala-Värtsilä located in the municipality of Tohmajärvi, with more than 1 million annual border crossings.

The region can, however, be characterized as a typical new immigration destination with relatively little experience of international migrants. Rural communities in the region have historically been ethnically homogeneous, except for a group of Karelian refugees stemming from areas annexed to the Soviet Union after World War II. North Karelia differs from typical European labor migration destinations in the respect that it lacks a labor-intensive “pull-factor” for immigration (Rye and Holm Slettebak 2020). Currently, about half of the immigrants living in North Karelia are of Russian origin, as the fall of the Iron Curtain has enabled cross-border mobilities, including student exchanges and intercultural marriages (Davydova 2009; Pöllänen 2013).

Of the 160,000 inhabitants of North Karelia, 77,000 live in the regional center of Joensuu. The University of Eastern Finland and the North Karelia University of Applied Sciences are both located in Joensuu. In general, North Karelia suffers from a simultaneous relatively high unemployment rate, and a labor shortage in many sectors because the aging population has led to a smaller available work force. Currently, the elderly dependency ratio in North Karelia is as high as 34.89 (OECD 2017, p. 115). According to population projections, rural municipalities in eastern Finland will continue to have the oldest age structures among the Nordic countries (Grunfelder et al., 2018, p. 32), which implies an increasing demand for health care and other services. Maintaining living standards will therefore depend upon increasing migration and productivity.

At 7.3%, Finland has the lowest share of population born in a foreign country amongst the Nordic countries (Statistics Finland 2020). In line with trends in other Nordic countries, the capital region of Helsinki attracts the highest number of foreigners, including Russian-speakers whose share of Helsinki’s population is 2.8 percent (Varjonen et al., 2017, p. 12). Russians are the most important group of immigrants in North Karelia, making up 44% of 3 774 foreign citizens (Joensuun kaupungin selvitys 2020). Additionally, 2 369 North Karelians have dual citizenship, typically Finnish and Russian. Russian-speakers are both present and visible, for example, in everyday-life in the North Karelian municipality of Tohmajärvi where the interviews were conducted. In Tohmajärvi, Russian-speakers comprise no less than 4% of the municipality’s population (Varjonen et al., 2017, p. 12). Therefore, it can be argued that in the eastern Finnish countryside, Russian-speakers serve to portray immigrants in general. Russian immigration and immigration in general to the sparsely populated areas of eastern Finland is a gendered phenomenon, as females are over-represented (Karlsdóttir et al., 2018, p. 38). However, immigrants are a more diverse group consisting of labor, family and educational migrants, refugees, and asylum seekers from various backgrounds (Karlsdóttir et al., 2018, p. 26).

### Conceptual Framework

#### Resilience and Vitality Policy in the Finnish Border Area

*Resilience* is a concept which has been adopted from environmental studies to the social sciences, and which has been theorized and utilized in several ways. In rural studies, the interest has been on the capacity of rural communities to cope with economic and social transformations (e.g., shrinking populations and the restructuring of traditional rural industries), and their ability to adapt to these changing circumstances and recover from crisis (McManus et al., 2012; Kotilainen et al., 2015; Søholt et al., 2018). Issues of trust, sense of community, social inclusion, welfare, networks, and participation are all seen as vital factors for building local resilience (Søholt et al., 2018, p. 222). Therefore, in the case of immigrants, the crucial point is whether they are valued primarily for contributing to regional economic development, as a mobile input into the local labor market, or whether they are given roles as equal co-producers of local resilience. In the case of new immigration destinations, local decision-makers and other key actors may accept immigrants only as workers in sectors which are not attractive to the local population, or as entrepreneurs who create local businesses (Søholt et al., 2018). When immigrants are not welcomed as integrated members of communities, their contribution to local resilience may remain low (Søholt et al., 2018). In some cases, however, immigrants themselves do not perceive the local community as the only framework of their social, cultural and economic activities. For example, immigrants living in border areas tend to have transnational connections and responsibilities on both sides of the border.

In Finland, municipalities are responsible for promoting resilience at the local level. The municipalities of North Karelia have adapted a new approach as part of their municipal strategies. This approach emphasizes the importance of public, private and third sector networks in rural development. The new *vitality policy* calls on the municipalities “… to build their strategies based on unique regional resources as well as to holistically involve other local organizations and individual citizens (communality) and to incorporate different policy sectors in their (rural) development work” (Makkonen and Kahila 2020). In practice, these vitality policies put an emphasis on raising employment rates for initiating a circle of positive development, including increasing municipal tax revenue, improving municipal services and living environments, and attracting new inhabitants to the local community. In the municipality of Tohmajärvi, its border location and an inflow of immigrants have been identified as potential and unique sources of vitality.

Within border research, the resilience of border areas has been explored as an ambivalent combination of geopolitical threads and a resource for regional development. According to Prokkola (2019), an open border “entails proximity to foreign markets and labor, the possibility to take advantage of cost differentials, the diffusion and stimulation of new knowledge and ideas as well as new regional identities and brands (Sohn, 2014). Some cross-border regions are also argued to serve as ‘innovative platforms for multidimensional integration processes, which are needed for more sustainable ways of living’ (Blatter 2004, p. 402)”. After the fall of the Iron Curtain and the enlargement of the EU, border regions which have traditionally been seen as peripheral and less developed, have become recognized as “motors of development” (Blatter 2004; Sohn 2014). This “window of opportunity” has been identified in eastern Finland since the collapse of the Soviet Union (Eskelinen and Zimin 2004; Izotov and Laine 2012). On one hand, migrants are seen as potential workers, especially in sectors which suffer from labor shortages. In eastern Finland, the care sector is a typical example (Jokinen and Jakonen 2011; Könönen 2011). However, in line with findings from other countries (Rye and Holm Slettebak 2020), research indicates that urban regions in Finland have benefited more from immigration than remote rural regions (Saartenoja 2010; Sarvimäki & Hämäläinen 2010; Reini 2012; Poutvaara 2019). On the other hand, migration raises the issue of whether and how newcomers are integrated as active members of the local society and support its overall resilience and vitality, or whether they tend face precarisation as workers in irregular employment combined with periods of unemployment (Precarias a la deriva 2009; Könönen 2015; Pöllänen and Davydova-Minguet 2017). The latter line of development may not be experienced as a problem by the migrants in the short term, but it can undermine the overall development and vitality of local communities in the long run. Seen from the perspective of Russian migrants, the decision to move to the neighboring country does not typically sever connections with their homesteads, and they tend to maintain regular transnational contact with their relatives and other social networks. As a result, they can also contribute to the vitality of their places of origin. In eastern Finland, this aspect of immigration is of special interest since most Russian immigrants come from areas close to the border.

#### Integration and Transnationalism

The concept of integration has developed in European discussions on governance and the research of migration, in opposition to the concept of assimilation (Saukkonen 2020, p. 17–22). Integration cuts across various aspects of migrants’ accommodation in the new society, and the political measures taken in order to enhance it. It concerns migrants’ integration into the economic, social, cultural and political spheres of society, but also today, integration is considered as a process that includes the society as a whole, and therefore the conversations and research concentrate on the discrimination that migrants face, how different policies affect migrants’ inclusion, and how the public perceives migrants and immigration (Saukkonen 2020; Sotkasiira 2018a; 2018b).

In Finland, the integration-related discussion, research, legislation and policies are co-developed with the process of the Europeanization of Finland (Puuronen 2004). The current Act on the Promotion of Integration (2010) sees integration as an interactive development between an immigrant and society, which aims at supporting the immigrant in developing skills required in society and working life while supporting the maintenance of his or her own language and culture (Yijälä and Luoma 2018, p. 48). (Hiitola et al. 2018, p. 14) state that in Finland, integration functions primarily as an administrative concept, the purpose of which is to promote equality and positive social interaction. Even though it is a loose administrative concept, in the public discussion it is often narrowed down to linguistic and occupational schooling of immigrants and their integration into the labor market (Hiitola et al., 2018, see also; Saukkonen 2020). In a broad sense, the concept of integration refers to the whole process by which an immigrant finds his or her place in society (Saukkonen 2020; Hiitola et al., 2018, p. 16). However, integration is strongly associated with the idea of guiding and helping the immigrant to adapt and settle (see Sotkasiira 2018a; Sotkasiira, 2018b; Haverinen 2018; Hiitola et al., 2018).

Whereas integration is based on the views of the host societies, and has been criticized for its container-thinking, the concept of transnationalism focuses on migrants’ social, political, cultural and economic networks which transcend the borders of nation-states (Martikainen et al., 2006; Huttunen 2002, p. 44; Martikainen and Haikkola 2010, p. 15). From the perspective of the vitality of rural border areas, integrationalist and transnationalist perspectives on migration should inevitably complement each other (see Levitt and Glick Schiller 2004). Especially, transnational relations do not exclude the desire of immigrants to integrate into their new surroundings and communities, and can be seen as a resource to increase the vitality of the region. On the other hand, local labor market, economies, businesses, legislation and different integrational measures, as well as the attitudes of the majority population frame everyday lives of immigrants, making it livable or not, and affecting their decision to stay or to leave the region. Immigrants’ integration into society and their interaction with the surrounding community can be seen, for example, in their participation in NGOs and voluntary activities, decision-making, voting activity, through social relations or owning property, or from their participation in hobbies.

In migration research, immigrants’ transnational connections and their impact on their new home societies have been an object of long-term research (e.g., Flemmen and Lotherington 2008; Flynn and Kay 2017). From the point of view of rural vitality, transnational and especially cross-border connections can have positive consequences. In the Russian-Finnish context, attention has been concentrated on Russian immigrant women’s transnational families, care, and their everyday life (Siim 2007; Pöllänen 2013; Pöllänen and Davydova-Minguet 2017; Davydova-Minguet and Pöllänen 2020). In our previous studies, we have revealed that in the context of North Karelian rural areas, precarious employment and the everyday lives of Russian-speaking women differ from that seen in urban areas. When combined with the close proximity of the border, it in fact enables vivid transnational family connections and care (see Pöllänen and Davydova-Minguet 2017; Davydova-Minguet and Pöllänen 2020).

## Materials and Methods

The empirical part of the present article is based on two data sets: text material obtained from the *Karjalainen* regional newspaper, and ethnographic interviews of Russian-speaking immigrant women living in the border region. By using the two data sets, we approach the role of immigration in the vitality of North Karelia from the points of view of the region’s Finnish-speaking key actors, and also the immigrants themselves.

### Newspaper Material Collection and Analysis

#### Data Collection

The *Karjalainen* newspaper (The Karelian) has been issued since 1847, and is the leading daily newspaper in North Karelia. Although it contains foreign, domestic, regional and local news, it has the characteristics of a regional newspaper. It represents traditional printed media which competes with free newspapers, internet news, and social media. According to the *Karjalainen*’s webpages, it reaches about 80 percent of the North Karelian population, and is currently available in both printed and digital forms. All issues published between 1847 and 2020 have been digitalized and stored in a digital archive that is available to subscribers.

Articles commenting on the role of immigration in North Karelian vitality were searched for systematically from the digital archive. As the focus of the present study is on current debate, articles published between 2011 and 2019 were selected for scrutiny. On the first round of data gathering, all articles containing the search term maahanm* [Finnish original–immigra*] were collected and stored in digital form. In the second phase, based on their content and author, the items were classified into categories of foreign, domestic, and regional and local news. Generally, the *Karjalainen* publishes more world and domestic news than regional or local news on immigration. The number of foreign and domestic news items was highest in 2015 during the war in Syria, as more than 32,000 asylum seekers arrived in Finland (Karlsdóttir et al., 2018, p. 24). Recently, the focus of domestic articles has been on documenting the rising support for figures of the Finnish anti-immigration populist party Perussuomalaiset *(the Finns Party)*. The peak year of regional and local news about immigration was 2012, when a group of Somalian refugees arriving in North Karelia hit the headlines (Sotkasiira and Haverinen 2016).

Immigration in North Karelia is commented on in 641 items in total. They consist of editorials, columns, interviews, news, reports, and the opinions of readers. The voice of the newspaper is represented in editorials and columns, and in the selection of other types of articles as well. Finding the balance between good journalism and meeting readers’ expectations is not a simple task, as the newspaper has competitors which provide news for free. In Finland as well as in many other European countries, the media discussion of migration is both polarized and politicized (Horsti, 2015). In 2015, when the discussion was heated, the Editor of the *Karjalainen* replied to critics who accused the newspaper of being biased because readers’ (negative) opinions on immigration had not been published, stating that the freedom of speech does not mean that racist opinions deserve to be printed (*Karjalainen* 2015a, p. 2). For immigrants themselves, news provided by the internet and social media in their own language are more easily available (Sotkasiira 2017). The *Karjalainen* newspaper is targeted implicitly at a Finnish speaking audience, and immigrants are not intended to be amongst its readers.

#### Analysis Method

Qualitative content analysis using an inductive approach was utilized in the analysis of the newspaper material (Berelson, 1952; Berg, 2001; Neuendorf, 2002; Krippendorff, 2004). The text mass of 641 items was reduced by coding the contents of articles, and identifying those which discussed the role of immigration in the vitality of North Karelia/North Karelian rural localities. Examples of the codes of the category “articles concerned with vitality” in the first stage of coding were “labor shortage”, “declining population of the region”, “aging and retiring population”, “need of enterprises to become international”, “immigrants as rural entrepreneurs”, “immigrants working as highly educated experts”, etc. After the first round of coding, and re-reading the articles, a total of 105 items discussed the area of “vitality” from some perspective.

The 105 articles were analyzed in the following stages. The first stage utilized the above coding of the contents of the articles (deconstruction) to isolate specific sub-groups. In the second stage, the original articles were re-read, and the coding system was checked and modified (recontextualization). During the re-reading process, the contents of the original texts were re-evaluated and in some cases their categories were changed. To create fewer categories, codes with similar contents were combined. After that, the contents of the news items were classified in further different categories (categorization). The material is presented in four main themes (Table 1). The analysis stays close to the text using the words and phrases used in the articles (author’s own translations), but aims at understanding the underlying meaning of the text.

**TABLE 1:** The analysis process of comments concerned with the role of immigration in the vitality of North Karelia.

Code	Category	Theme
Declining population	Demographic factors	“Despite the immigration, the region is shrinking, and the countryside is declining”
Aging and retiring population		
Too few babies being born		
Negative population forecasts		
Slightly positive migration figures		
Labor shortage	Labor market needs	“Targeted, controlled immigration is needed by the regional and rural labor markets”
Shortage of appropriate labor force		
Need for Russian speaking workers		
Shortage of cleaners and nurses		
Need for more entrepreneurship		
Need for educated labor		
Immigrants are/can be educated		
Immigrants are willing to work		
Foreign students are wanted		
Businesses need to become international	Internationalization	“Mobility and multiculturality make the region more attractive for people and businesses”
Capital is mobile and the region must be attractive for domestic and foreign companies		
People are mobile in the mobility era		
Immigrants enrich the region socially and culturally	Multiculturality	
Immigrants are part of the street view		
How to make immigrants and their children stay?	Integration	“Wellbeing and integration of immigrants generates wellbeing for the region”
Wellbeing of immigrants generates wellbeing for the region		

The media analysis on the perceived role of immigration in the vitality of North Karelia focuses on the following issues: 1) the participants in regional and local discussions on immigration, and 2) the aspects of vitality that are given emphasis in the public discussion: whether the discussants value immigration primarily as a contribution to regional economic development, or whether immigrants are given broader roles as co-producers of local resilience. In the interpretation of the material (compilation), attention is especially paid to *who* is talking about immigration, *what* is being said, and *how*.

### Ethnographic Research

The collection of interview material was embedded in the methodological tradition of the ethnography of everyday life (Vila 2003; Jokinen 2005; Passerini et al., 2007; see also; Flynn and Kay 2017). In the analysis of the interviews, we were interested in how immigration impacts the vitality of rural areas when examined from the point of view of immigrant women, and how media discussions coincide with the experiences of Russian immigrant women. We paid special attention to women’s transnational ties, and their meaning in making border regions attractive and livable.

Everyday ethnography has two dimensions. First, it concerns the object of interest, namely people’s everyday life. Secondly, it refers to specific research methods, including data collection, analysis and ethnographic writing. Everyday life can be studied only by participating in it, by being there, and drifting alongside the people (Junnilainen 2019). For the last twenty years, we have studied Russian immigrant women’s everyday life, mostly by using the methods of participant observation and ethnographic interviews (see Davydova 2009; Davydova and Pöllänen 2010; Pöllänen 2013; Davydova-Minguet and Pöllänen 2020). We approach our research field holistically, which means that in our analysis we raise such aspects that are felt to be important on a structural level. Our knowledge, approach and analysis of the field is therefore developing cumulatively from both newer and older datasets, as well as drawing from previous research.

The dataset for the current study was produced within our recent research project (2015–2017) on the perceptions of Russia in the border area of Eastern Finland, conducted in the Tohmajärvi region. The data consists of 21 in-depth interviews conducted with Russian-speaking dwellers of the border region in total. Most of the interviewees (16) were women. In this article we analyze the interviews of female participants, while our ethnographic notes, participant observation materials and interviews of male participants frame our understanding of the overall situation. The interviews lasted from 45 min to 3.5 h. The interviews were recorded and transcribed. Russian-speaking informants were interviewed in Russian, and material was analyzed in original language and translated into Finnish/English only when used as a citation. The informants were aged between 32 and 63 years old, with different levels of education, many with higher education degrees. Some of them had re-educated themselves for new professions in Finland. All the women had families, and some were married to Finns, some to Russians, some divorced, and some had children. The women had lived in Finland from anywhere between two to more than twenty years. All of them were in a precarious position on the labor market, although most of them spoke good Finnish.

The analysis of our interview data followed the principles of thematic analysis. We progressed by close reading (Pöysä 2010) the interviews from the angle of local resilience and vitality. In connection with the reading, we classified and coded the interview material thematically. The close reading and thematic classification were guided by the questions of how immigrants attach to the area, what do their transnational connections mean for their attachment to the region, and what are the other aspects of their lives that can impact on the resilience and vitality of the studied rural border region.

As researchers we always must take responsibility for our decisions and choices and consider their consequences for the informants. In this study, the common ethical rules of social sciences have been followed, the most important of which is to avoid causing harm to the informants (Kuula 2011; TENK 2019). The research material has been compiled from a relatively small community in North Karelia, and the informants could therefore become easily recognized. Therefore, we have avoided giving detailed descriptions of our informants in presenting the data and analysis, in order to protect their anonymity.

## Results

### Newspaper Discussions About the Role of Immigration in the Vitality of North Karelia

The discussion in the regional newspaper on immigration has been vivid and various contributors have participated in it. Several actors representing North Karelia’s business life, state authorities, rectors and researchers of the University of Eastern Finland (UEF) and the North Karelia University of Applied Sciences, experts and consultants in regional development, regional and municipal leaders, civic society actors, politicians, and also the newspaper in the form of editorials and columns, participated in discussions about the role of immigration in the vitality of North Karelia. In addition, some immigrant representatives featuring academics and activists of the local Joensuu District Multicultural Association commented on the issue. Overall, the immigrants’ voice is missing from the newspaper discussions on immigration, which are dominated by the Finnish-speaking “elite” of the region.

Table 1 shows how the comments were coded and categorized, and the four main themes identified from the discussions. During the period 2011–2019, problems related to the declining and aging population have been present every year. Labor shortage and the need to attract an appropriate foreign labor force is a more recent discussion which currently dominates the discussions in the newspaper. Notably, arguments supporting controlled labor migration started to gain ground after the Syrian war and the humanitarian crisis which increased the amount of asylum seekers seen in North Karelia.

The need for businesses to become more international, and any positive social and cultural effects of multiculturality are less often used arguments than factors related to the declining population and labor shortage. In Table 1, the categories of internationalization and multiculturality are merged under the same theme. Comments which argue the need to improve the overall well-being of immigrants, and the need to integrate them into Finnish society have been rare during the time under investigation. Four main themes emerged from the media texts, and are now discussed in more detail.

#### Despite the Immigration, the Region is Shrinking, and the Countryside is Declining

The first theme consists of remarks on negative population forecasts and slightly positive levels of migration in North Karelia. Every year, after the official population statistics are published, discouraging demographic facts such as an aging population and low fertility rates are pointed out. Typically, the head of the Regional Council of North Karelia and the town manager of Joensuu, “celebrate” the slightly positive migration figures (e.g., *Karjalainen* 2011a, p. 4; Karjalainen, 2012a, p. 8). However, other commentators including the Editor of the *Karjalainen*, argue that “*immigration will have no factual effect on population figures in rural locations*” (*Karjalainen* 2011b, p. 2), “*immigration does not solve the problem of population decline in North Karelia*” (*Karjalainen* 2015b, p. 2) and “*in spite of the immigration, the future population development will be negative*” (*Karjalainen* 2019a, p. 4). A professor of Human Geography predicts that in a pessimistic tone that despite immigration, North Karelian “*rural areas will be uninhabited in the future*” (*Karjalainen* 2014, p. 19). Therefore, it is seen that although migration figures are positive and immigration is argued to be crucial, the key actors of the region do not expect that immigration will positively influence the population figures of the region.

#### Targeted Immigration is needed by the Regional and Rural Labor Markets

Labor shortage is a source of worry for private and public sector employees, politicians, researchers, university representatives, municipal leaders, employment authorities, and the newspaper itself. Typically, the commentators on this topic support “targeted, controlled labor immigration” which meets the labor market needs. For example, the rector of the University of Eastern Finland supports targeted immigration to increase the number of students and workers in certain branches of education and businesses (*Karjalainen* 2019b, p. 8). From the texts, it can be seen that more workers are required in the primary sector, low paid service and industrial jobs, tourism, and health care. As the Manager of the North Karelian Chamber of Commerce argues, “*the labor shortage is unbearable without immigrants who would be willing to work”* (*Karjalainen* 2017, p. 28; Karjalainen, 2018, p. 31). The need for Russian speaking staff is frequently mentioned (*Karjalainen* 2011c, p. 2; Karjalainen, 2012c, p. 4), and rural municipalities including Tohmajärvi encourage immigrants to set up new enterprises which would bring new livelihoods to rural places (*Karjalainen* 2012b, p. 4; Karjalainen, 2013a, p. 3). In the region where the unemployment rate is rather high, around 15% of the workforce, the discussion in the newspaper about the targeted immigration of the workforce tends to be over-simplifying, somehow concealing the lived experiences of immigrants who have to find jobs in this area. The latest research on the labor market demands in rural areas in North Karelia shows that there are shortages in highly educated work force in health and social sectors (Jolkkonen and Lemponen 2016).

#### Mobility and Multiculturality Make the Region More Attractive for People and Businesses

Discussions about internationalization in the *Karjalainen* newspaper point out that we live in a mobility era which encompasses the movement of people, capital, goods, and ideas. The Editor, the Manager of the North Karelian Chamber of Commerce, representatives of the Regional Council of North Karelia, professors and researchers argue that mobility is necessary, especially if the region aims to survive in the internationalized world. For example, one reference to the United States argues that “*the more diverse a region is, the more potential there is for new innovation and growth*” (*Karjalainen* 2014, p. 19). Researchers, immigrant representatives (who are often academics), the local Joensuu District Multicultural Association that represents people from various backgrounds, and the Editor present multiculturality as a valuable resource which enriches the region and makes it more attractive for people and businesses (Karjalainen, 2012a, p. 17; Karjalainen, 2013b, p. 16; Karjalainen, 2014b, p. 2). The commentators highlight the role of international students and academics who increase the knowledge, expertize, and social capital of the region. Clearly, by stressing the positive impact of cultural and ethnic diversity, the perspectives of the academic immigrants talking in the newspaper introduce a new facet to the discussion about vitality.

#### Wellbeing of Immigrants Generates Wellbeing for the Region

Whereas in most texts immigrants are valued primarily for contributing to the region’s economy and the material conditions of the native population, a minority of articles see immigrants as having potential for promoting regional or local resilience, if they are included and integrated in Finnish society. The local Multicultural Association, immigration authorities, researchers, and individual citizens emphasize that immigrants are “*already a natural part of the street view*” (*Karjalainen* 2011a, p. 4). They are, however, worried about the hostile attitudes that immigrants encounter. “*A civilized country should not treat immigrants as stranger*s”, argues a civic society actor, “*we need immigrants to build the Finnish society*” (*Karjalainen* 2011a, p. 4). The Editor pays particular attention to highly educated immigrants who end up leaving the region because they are discriminated against in the labor markets: *“Do we let immigrants find their place in society? There are well-educated immigrants who have lived here a long time, and we keep on treating them as outsiders”* (*Karjalainen* 2013c, p. 2). Experts fear a brain-drain and an exodus of second-generation immigrants, as better job opportunities and a more tolerant atmosphere elsewhere may tempt them to move out of the region.

#### Interpretation of the Findings

The central finding of the newspaper material analysis is that the “elite” of North Karelia mainly understands the significance of immigration in a narrow sense, as an input to the region’s economy, rather than in terms of increasing cultural and ethnic diversity (see also Søholt et al., 2018). Immigrants are supposed to be “targeted” labor migrants who boost the economy and service production, and thereby improve the material conditions of the *native Finnish population* (see Könönen 2015). In line with findings in other European countries, labor migration is supported as it helps to maintain the welfare of the citizens of European welfare states (e.g., Bommes 2012). Few commentators criticize this narrow economy-based approach and argue for the need to understand immigrants in a broader role as a natural part of the region’s diverse population and included members of communities. Overall, the key actors of North Karelia talk about immigrants as inputs to the region’s vitality rather than as equal co-producers of local resilience which benefits immigrants as well as the native population. With the exception of the representatives of the local Multicultural Association, immigrants themselves do not participate in the newspaper discussions, which suggests that immigrants are outsiders in the discussion of the vitality of the region, where they live their everyday lives.

### Findings: Interview Material

#### “Being at Home” on Both Sides of the Border

Russian-speaking immigrants have come to eastern Finnish rural areas predominantly as wife-migrants or so called return migrants (Soviet and Russian citizens of Finnish origin who during 1991–2016 had the right to move to Finland on the basis of their ethnicity: see Davydova, 2009). Most of the informants of our study had moved to North Karelia from the adjacent Russian Republic of Karelia, or other nearby areas of Russia. Women of Russian origin are the most common foreign-born wives of Finnish men, and especially common in this region. The marriage-based immigration channel is based on the intensive and short-distance travels to the Russian side of the border by Finnish men who live in the border areas. Russian-Finnish relationships and marriages occur typically between persons who live close to the border, and eventually affect broader kin and friendship networks.

As another category of immigrants, Soviet and later Russian citizens of Finnish descent have traditionally lived in Russian territories close to Finland, and their migration to Finland also involves broader families and friendships. So, the immigration of Russian-speakers to Eastern Finnish rural localities is conditioned by available migration channels and already existing *trans*-local patterns and networks. Subsequently, this immigration strengthens and expands already existing networks, and contributes to the formation of further migration chains. When viewed overall, it can be said that these cross-border connections form a sense of “being at home” among Russian-speaking dwellers in Finnish border areas. This is manifested in the following interview excerpt:

Interviewer: How is the proximity of the border manifested in your everyday life? Respondent: Probably through tourists. There are many Russian tourists. Maybe berry pickers in the summer. It feels like being in Russia, because everywhere you can hear the Russian language. There are many people who go to Russia to refuel their cars, probably because here gasoline is much more expensive, almost two times, and it is cheaper there. Maybe the Finns somehow feel their proximity to the border as insecurity, but I don't feel that. I don’t notice the border at all - when I cross it, I end up at home, you just drive over and are again at home (laughs) (Informant, born 1961).

According to (Saartenoja 2010, p. 27) the way of understanding and experiencing *place* is different for different migrant groups. The relationship with the local place is dependent on the migrants’ background, and how easy or difficult it is for migrants to adopt or settle down in rural area in Finland. According to previous studies (e.g., Saartenoja 2010), it seems that if a migrant comes from an urban area or big city, then the silence and emptiness of Finnish rural areas might become a source of anxiety. However, for Russian-speaking migrants, Eastern Finnish rural areas form a suitable and comfortable place to settle because of their proximity to the border and their places of origin. Here they can effectively maintain transnational connections. In the following excerpt, the informant tells about her transnational way of life:

Interviewer: What is Russia for you? Respondent: Russia is everything to me. My children live there, my parents live there, I was born there. And my husband and I bought a house very close to the border, we go there every week, we have an apartment there in Sortavala, where I come from (Woman, born 1955).

Saartenoja (2010) points out that marriage helps one to adopt to local conditions, even in terms of living in rural areas. In Finnish rural areas, most of the migrants are women, and many are married with Finnish husbands. For example, in the Kainuu region there are 30% more migrant women than migrant men. For wife migrants, it is easier to settle down in rural areas than for other migrants because they have their family as a support network and marriage also gives them higher status (Saartenoja 2010, p. 30–31). Additionally, marriage with a Finnish man can mean a better economic position, in terms of owning a property. Marriage can also help people to access social and health services and get jobs, especially if the husband is an entrepreneur whose business is connected with Russia (see also Saartenoja 2010, p. 30–31). In the following excerpt, the informant describes her husband as being “too good”, and also her attachment to the border region:

Respondent: Just a very good person for me - too good (laughs). In my youth, the fortuneteller guessed that my life would change after 45. And it seems to be true. I also have real estate here. I have such a husband. Such an honest, decent person, he shares half of everything with me (laughs). Although this rarely happens here.

Interviewer: Would you like to live somewhere else? Maybe there is some other city here in Finland? Respondent: Of course. But we now live here, and travel to the border. We have moved many times. We started to live in Tohmajärvi, then moved to Joensuu. We lived there for 5 years because he had a job there. And I studied there at the University. And then, my husband says: I have a pension, I'm so tired of the city, let’s go closer to the lake, fishing and hunting. We bought a house. And now he is renovating it, improving it, building everything. Of course, I wanted to be in the city, but no problem, we have a car and he can drive me there (Woman, born 1955).

It can be concluded that the integration of Russian-speaking female immigrants into Finnish border areas happens predominantly due to a close proximity to the check point on the border, which reciprocally enables their transnational ties. In immigrants’ everyday lives, the sense of “being at home” raises from the possibility to smoothly combine “old” and “new” places. This aspect seems to be lacking in the newspaper discourse.

#### Russian Speakers Advance Resilience and Vitality of Border Regions

The majority of Russian-speakers moving to rural areas experience difficulties in finding jobs. Employment courses for immigrants organized by public authorities seem to be an essential channel through which to get a job, to form networks with other immigrants, and which form a considerable part of their precarious everyday lives. The most probable employment can be found in services aimed at Russian tourists, but which involve short-term and part-time employment periods. Nevertheless, when combined with lower expenses, possibilities for picking berries and mushrooms, hunting and growing their own vegetables, and buying cheaper goods in Russia, it provides many immigrants with an opportunity for a relatively satisfactory economic well-being. In the interviews, people present themselves as active actors who search for and find jobs despite the obstacles connected with rurality, and creatively use the opportunities of living in the countryside.

Interviewer: Would you like to live somewhere else? Respondent: No, I like Finland. I would not want to live anywhere else. I like Finland, I have many friends here, I already have a lot of connections with Finland. I told you, not only am I a fisherwoman, I am a hunter. Interviewer: So you have a gun? Respondent: I shoot hares that foolishly run out at me. It was my Finnish husband who implanted this in me, such a love for all of this. I lived in the middle of the forest, and there were no other hobbies. There, you had to either accept his hobbies, or stay at home. But since I cannot sit at home, the first course I took in Finland was to get a hunting ticket. With three big dictionaries, I passed the exam (Woman, born 1960).

According to our ethnographic study it is clear that Russian immigrant women also have close relationships with Finnish speaking local dwellers in the Tohmajärvi border region. Local people see the Russian speakers as benefiting local everyday life, and also the economic life of the region. Russian speakers can also be good news for the service sector because of their language and social skills. This resonates with the viewpoints presented in the newspaper material.

The trump card of Russian-speakers on the local labor market is their ethno-cultural capital which consists of their native proficiency in the Russian language, their cultural knowledge, and their local and *trans*-local social networks (see Könönen 2011; Davydova 2012). The development strategy of the municipality of Tohmajärvi (Municipal Strategy Action Plan, 2020) highlights several activities related to the border and Russia as being a strength of the municipality, which also means job opportunities for local Russian-speakers. According to our data and previous research, it can be noticed that the most likely jobs can be found either in grocery stores, private nursing homes, as an interpreter, a language teacher, or in tourism-related activities. The ethno-cultural capital of migrants can be utilized to serve both Russian-speaking local dwellers and Russian-speaking border crossers, and is also useful for cross-border economic activities. Martin et al. (2013), p. 52 consider that from the point of view of the export industry, the language skills of immigrants may be an advantage for companies and promote their exports. In Finnish-Russian border areas, migrants’ ethno-cultural capital benefits both the regional vitality, and also the immigrants’ welfare. Here, a Russian-speaking immigrant woman describes her job as the hostess of a holiday village in North Karelia:

Respondent: The Russian language is my bread for me - it feeds me now, one might say. If I didn’t know Russian, I wouldn’t work here. I have been working here for nine years. When I came here to work, we had three ladies here: two Finns and me. For some reason, in the end, only I remained. You have more clients with Russian language, since we are close to the border. And everyone who comes asks right away: where is She? But I get tired more than anyone else, because I need to speak in both languages, in one, in the other, and in a third language (Woman, born 1962).

It can therefore be concluded that the transnational networks formed by immigration benefit regional development, local business life and entrepreneurs not only by providing them with the ethno-cultural capital of immigrants, but also by attracting more clients from both the Russian and Finnish sides of the border, thus enhancing vitality and resilience of the border region.

#### Russian Speaking Women as a Demographic and Care Resource

“Keeping rural areas alive” entails not only an involvement in production, but also in re-production. Many of our interviewees have children who were either born in Finland or who come from previous marriages in Russia. In Finnish rural localities, these children attend schools, and use other public and private services. So, immigrant women can be seen as a demographic resource for shrinking rural regions. Even if the local media discussion (see *Karjalainen*) does not recognize migrant women as a deliverance, on the everyday level, their presence provides at least a plaster for rural vitality.

Interviewer: What do you think the concept of the welfare state means? Respondent: A state that can and wants to provide for those who can no longer provide for themselves. These are children, old people. Naturally, the welfare state cannot function without the people who work for this state. I don’t blame Finland for high taxes, I pay them and don’t cry, because I understand that no old man will be abandoned because of these taxes, and my child goes to a free school, and he gets free meal there, and he studies in good conditions, in a safe environment. Education has been provided for him, and if anything happens, we will get to a hospital and so on. It is such a mutual responsibility–a worker who can work, must work for the state, helping the state. In turn, the state will help those people who need help.

Interviewer: Do you use such services? Respondent: Yes, health care, school, naturally.

Interviewer: Are they far or close to the place where you live? Respondent: Close. Everything is absolutely accessible. Interviewer: Which of these services do you consider the most important? Respondent: Probably education and health services. I would put them side by side, because both are very important for a person (Woman, born 1979).

At the same time, from the point of view of demographic development in remote rural areas, Russian-speaking immigrant women are a valuable resource for the region's care labor market, which needs precarious care labor to look after the region's aging population (see Jokinen and Jakonen 2011; Könönen 2011). In Tohmajärvi, the provision of care has shifted from the public sector to private business, which needs a flexible, low-paid labor force. In addition, as there are Russian-speakers of all ages in Finland and some are already in need of care services, the ethno-cultural capital of migrants is needed, and might become a key asset in care workplaces in the future.

Russian-speaking immigrant women participate in both formal (paid) and informal family care. They have family members in both Finland and Russia, and have care duties and obligations in both countries. In Russian migrant women’s lives, transnationalism means large array of practices ranging from media consumption to care relationships (Pöllänen and Davydova-Minguet 2017; Davydova-Minguet et al., 2019).

Interviewer: How has your life changed after moving to Finland? Respondent: The first years were hard, because they were there, and I was here. But I went there every week - I still drive, and I continue to drive there. At first I earned money, I was a breadwinner for a family [but it was] so fragmented: children in one place, mother in the other. My heart was divided into two parts. One part in Russia, and the other in Finland. So I live, half of me here, half there. <…> And about my mother. It is now difficult even to bring her to Finland, because she is Ukrainian and can get a visa only in Kiev. And my mother is very sick, she cannot go to Kiev, especially not to wait for a visa. Therefore, my mother cannot come here (Woman, born 1960).

It seems that care has a significant role in the everyday lives of Russian immigrant women. Contrary to the newspaper discourse which anticipates immigrants as a care resource in formal institutional care, family care is central in the everyday lives of the interviewed women. However, family care is also essential in enhancing the resilience of the border region, as women are responsible for the care of their parents and parents-in-law, as well as the members of their extended families on both sides of the border. Thus, the transnational dimension of migrants’ care is of high importance for both themselves and their families (See also Pöllänen and Davydova-Minguet 2017).

## Discussion

In the present study, we explored the interpretations of the role of immigration in enhancing the vitality of the Finnish-Russian border region of North Karelia, which has had relatively little experience of international migrants. We investigated how the impact of immigration is presented in the regional *Karjalainen* newspaper, and how the vitality of the border region can be perceived through the interviews of female Russian immigrants. The interview material was analyzed through the prism of rural vitality policy, which stresses the unique resources of a place and calls for a holistic and inclusive approach to rural development (Makkonen and Kahila 2020). We were interested in whether immigrants are valued only as contributors to economic development in regional media (as they tend to be in remote rural environments in many countries), or whether they are presented in more broader roles as co-producers of local resilience (see Søholt et al., 2018). Furthermore, our thinking was guided by the aspiration to combine both integrationalist and transnationalist perspectives on immigrants’ lives and processes associated with migration. Notably, the integrationalist stance has dominated Finnish research and media conversation on immigration, yet in the 2000s, the societal consensus on immigration has shifted from the promotion of ethnic and humanitarian immigration, toward advancing labor migration (see Lepola 2000; Könönen 2015).

The same stance can be identified in the regional media, where the key actors of the region argue from a narrow perspective of “targeted labor migration” that would help to fill jobs and maintain businesses in remote areas. This media discussion about immigrants as a mobile labor force that boosts the poor population figures, lacks the idea of immigrants as members of communities who contribute to the regional and local resilience. The lived experiences and narratives of immigrants show that they see this differently. Similarly, in most newspaper comments concerning the role of immigration in the internationalization and multiculturality of the region, immigrants are seen as a resource which makes the region more attractive for people and businesses. However, against expectations, the newspaper discussions in this new immigration destination also include comments, mainly from the university personnel and educated immigrants themselves who represent the local Multicultural Association, which stress the cultural and ethnic diversity of migrants as a positive phenomenon, not only in terms of improving the economy and material conditions of the native population. In some comments, immigrants are seen as a natural part of the region’s population who diversify the skills and capacities of communities, and thus form potential for promoting regional or local resilience. Yet, the Russian immigrant women themselves living in rural localities do not participate in the discussion about the vitality of rural communities, which indicates that they are not fully recognized as contributors of resilience in North Karelia. Furthermore, Russian women remain “invisible” in the discussion on rural development.

As a further observation, the majority of immigrants who have settled down in the North Karelian countryside have arrived through other migration channels, such as family ties or marriage migration, the re-migration of persons of Finnish ethnic background, and through humanitarian migration. For these migrants, their integration into the labor market and local communities differs from the idealized pattern of labor migration, where migrants are expected to fill existing vacant positions, e.g., in farms or IT-companies. The analysis of the data shows that for Russian immigrant women, the rural border area of North Karelia forms a livable environment, where the closeness of the border enables women’s participation in the local precarious labor market. The jobs that women end up doing are usually not those which suffer from labor shortages, but are often based on women’s ethno-cultural knowledge and their networks. The interview data also shows that in rural areas, internationalization mostly occurs on the level of lived everyday lives, and especially in border localities it is based on transnational connections. Mastering the Russian language and culture has become an asset for those who speak Finnish fluently, and can be utilized by them in service or business sectors. For many though, the Russian language can restrict their possibilities and contacts with the Finnish speaking local population. Although multiculturality is often presented as an asset in official statements in public discussion, a lived multiculturality can have many more dimensions. The interviewed women emphasize their multilevel connections to the region where they live, and these include family and friendship relations which tie them to places on both sides of the border. Thus, the proximity of the border and the ease of its crossing forms an important factor that attaches them to these rural places.

On the basis of our interview data, it can be argued that the narrow understanding of integration as simply acquiring Finnish language skills and participation in the labor market which is characteristic of the administrative use of this concept, does not meet immigrant women’s own experiences of “being at home” in the border region. Furthermore, it seems that the easier the maintenance of transnational ties is, the smoother the integration process is, which in turn boosts the vitality and resilience of the region.

In this article, we have combined two approaches of rural studies and migration studies in order to investigate the role of immigration in rural development on the Finnish-Russian border. Our findings are in line with previous research in rural studies, namely that immigration is mainly understood as an asset to rural locations which suffer from a labor shortage, population decline and aging. This is the way that immigration has been approached and conceptualized in previous research (e.g., Rye and Holm Slettebak 2020; Kasimis et al., 2010) and how it is perceived in the regional newspaper in North Karelia. However, our study has demonstrated that in the context of proximity and neighborhood with the places of origin of migrants, their patterns of migration do not fully coincide with the expectations concerning their impact on the rural labor market. In these circumstances, the vitality of the rural border region should be approached from a more holistic and transnational perspective (e.g., Flynn and Kay 2017; Assmuth et al., 2018). The migrants’ attachment and commitment to the region should be seen as an important resource for the vitality and resilience of the rural region. This needs to be acknowledged in terms of broadening the official discourse on immigration, as well as the policies of integration and inclusion.

However, the context of the Finnish-Russian border is distinctive and creates factors that impact on the transnational realities of immigrants and local population. The geopolitical tensions between Russia and the EU, the growing mistrust toward Russia in Finland, and Russia’s mediatized identity politics create feelings of being “between the devil and the deep blue see” in Russian-speaking immigrants living in Finland (Oivo and Davydova-Minguet 2019). Consequently, this overall tensed situation and its impact on rural vitality and resilience prompts a need for further research.

## Data Availability

The raw data supporting the conclusions of this article will be made available by the authors, without undue reservation.
